# Supplementary material for: Assessing service availability and readiness to manage Chronic Respiratory Diseases (CRDs) in Bangladesh
Source: PLoS One. 2021 Mar 4;16(3):e0247700. doi: 10.1371/journal.pone.0247700 (PMC7932138; doi:10.1371/journal.pone.0247700)
Supplement: Attachment — Submitted filename: Response to Reviewers.docx [file pone.0247700.s003.docx]

**Response to Reviewers’ Comments to the Author (2^nd^ version)**

**Comments to the Author**
**REVIEWER #2:**

There are two versions of the revised paper: a first version commencing on page 19 immediately following the responses to reviewers and a second version headed ‘revised manuscript with track changes’ commencing on page 50.

Unfortunately, while the authors have inserted Table 1 into the revised first version of R1, there is still a discrepancy in lines 221 following in the first version:

The first version of the paper is as follows:

221 The strength of this study is that we explored the current scenario of CRDs management in

222 facilities throughout Bangladesh stratified by different levels. We explained it both in summarized

223 and disaggregated in all four domains according to WHO SARA Standard Tool. However, there

224 are few limitations such as we could not include all the sample health facilities due to incomplete

225 questionnaire and absence of proper interviewee. Also,changes over time could not be observed

226 as it is a cross-sectional study. The available data only reflects on the snapshot of availability and

227 readiness.


The second version of the paper has the following paragraph at the end of the discussion section in the same position as the paragraph above:

228 The strength of this study is that we explored the current scenario of CRDs management in

229 facilities throughout Bangladesh stratified by different levels. We explained it both in

230 summarized and disaggregated in all four domains according to WHO SARA Standard Tool.

231 However, there are few limitations such as we could not include all the sample health facilities

232 due to incomplete questionnaire and absence of proper interviewee. Data is based on reported

233 information from the interviewed staff member and was not confirmed by observation. Moreover,

234 we had to use same indicators for all level of facilities as there is no specific evidence

235 mentioning what services should be provided at which level. Also, changes over time could not

236 be observed as it is a cross-sectional study. The available data only reflects on the snapshot of

237 availability and readiness.

The second version, while not marked in track changes, contains the changes requested in the review, and should replace the same paragraph in the first version.

**Response:** Thank you so much for your feedback. We have addressed your concern and replaced the paragraph containing the changes in **line (228-235), at the end of the discussion in the revised manuscript without track change mode** as below-

“The strength of this study is that we explored the current scenario of CRDs management in facilities throughout Bangladesh stratified by different levels. We explained it both in summarized and disaggregated in all four domains according to WHO SARA Standard Tool. However, there are few limitations such as we could not include all the sample health facilities due to incomplete questionnaire and absence of proper interviewee. Data is based on reported information from the interviewed staff member and was not confirmed by observation. Moreover, we had to use same indicators for all level of facilities as there is no specific evidence mentioning what services should be provided at which level.”

**Responses to questions in the review of the first version of the paper**
Q1. Criteria applied to all levels of facilities.
I note the response to my question on different criteria for different levels of facilities: ‘we have used the same indicators for all levels of facilities’. However, even if facilities could not be distinguished on the basis of what services should be offered at what level, calculation of readiness should also take into account whether the facilities claims to offer the service.
However, the availability calculations in this paper appear to be calculated on the basis of all facilities at each level (Table 3 n values) .The WHO SARA guidelines explain that availability should only be calculated on the facilities offering the services. As the proportion of facilities offering the service (reported as availability) was much lower among MCWC and FWC/CCs, the calculation of readiness will be significantly altered.
See reference below:
<https://www.who.int/healthinfo/systems/SARA_Implementation_Guide_Chapter8.pdf?ua=1>
SARA Implementation guide: page 113
Step 3. Calculate the mean availability of each tracer item The mean availability of each tracer item is equal to the total number of facilities that have the tracer item available (i.e. value=1), divided by the total number of facilities OFFERING THE SERVICE, multiplied by 100 to get a percentage value.
The authors need to address this issue. If the authors choose not to use the SARA guidelines in the calculations, this should be explained; otherwise the calculations need to be repeated based only on facilities that claim that they offer CRD services.
Note that if the readiness scores are calculated based on facilities offering CRD services, then the comments in the discussion lines 212-217 may need to be revised – as the MCWC and FWC facilities also have low proportions who claim to offer CRD services.

**Response to Q1:** Thank you so much for your feedback. We have addressed the issue and re-analyzed our data. We rigorously did literature review and found that actually CRD services are provided upto Upazila (UHC) level only (Biswas *et al*) and MCWC, FWC provide only family planning services. So, we excluded MCWC (61) & USC,FWC, CC (214) from our analysis which made our sample size into **262.** We also edited these in methodology and inserted the following paragraph **(line 114-137**) **in the revised manuscript without track change mode** **-**

**“**In Bangladesh, public facilities are established according to its administrative units (division, district, upazila, union) where tertiary, secondary, and primary facilities are situated in the divisions (medical college hospitals), districts (DH, MCWC), upazila (UHC), union (FWC, CC) respectively [17]. As the number of population living in administrative units is different, so the population served by each facility is not defined. It may be mentioned that UHCs provide 31% of government health service and each of it serves 100,000 to 400,000 people depending on its size [18].

To identify the total number of hospitals at all three tiers in Bangladesh, the Bangladesh Health Facility Survey (BHFS) 2014, National Institute of Population Research and Training (NIPORT), Management Information System(MIS) Report of Directorate General of Health Services (DGHS), and records from director hospital administration DGHS were used (16). From this, we identified19,184 total hospitals and health centers in the country [19] .

From this total, the parent study, Service Availability and Readiness Assessment (SARA)

Survey for NCDs and Disability Service Delivery System in Bangladesh, selected 590 facilities using a stratified random sampling procedure according to administrative units and level of facilities **(Fig1).** CRD services are provided from tertiary level of health facilities upto upazila level health facilities in Bangladesh. [8] This includes UHCs, district hospitals, and medical college hospitals. Conversely, MCWCs and FWCs were excluded from this sub study since these facilities only offer family planning services, and not CRD services. Therefore, this sub study found a total of 273 facilities that provided CRD services. However, 11 more facilities were excluded from this sub study during the analysis of data. These facilities were excluded either because the questionnaires were incomplete or the hospitals were specialized in diseases other than CRD and were therefore irrelevant for this current study. The resulting sample size of 262 facilities is depicted in **Fig1.”**

**Fig 1: Sampling and study inclusion flow chart**

Total no. of health facilities in Bangladesh:19,184

Total no. of health facilities in sample during survey:590

- Tertiary hospital: 10
- District hospital (secondary): 60 (all)*
- MCWC (secondary): 61(all)
- UHC: 128 (30% of total UCHs in the country)
- FWC & CC:256 (2 facilities in correspondence to each UHC)
- NGO/Private: 75

No. of facilities excluded: 11

- Tertiary hospital: 2 (specialized in certain other disease like cancer and cardiovascular)
- District hospital:2 (incomplete questionnaire)
- UHC:4 (incomplete questionnaire)
- NGO/Private: 3 (incomplete questionnaire)

No. of facilities offered CRD services: 273

- Tertiary hospital: 10
- District hospital: 60
- UHC: 128
- NGO/Private: 75

No. of health facilities analyzed for this study: 262

- Tertiary hospital: 8
- District hospital: 58
- UHC: 124
- NGO/Private: 72

*N>B- UHC: Upazila Health Complex, MCWC: Maternal and Child Welfare Center, FWC: Family Welfare Center, CC: Community Clinic, NGO: Non Governmaent Organization.*

*rest of the district hospitals had been converted to medical college hospital (tertiary facilities)”

Also, we followed SARA guideline for calculating mean availability **(Table 2**). For example among 8 tertiary facilities, Pulmonologists are available in 6. So, the mean availability is 75.0%.

Then we calculated the readiness scores based on facilities offering CRD services as we have already excluded MCWC, FWC, CC **(Fig 2&Fig 3)**.

We also changed it in methodology in line **156-165** **in the revised manuscript without track change mode** like below-

“Mean availability and Service readiness were assessed in three stages: 1. Determining the mean availability of CRD service readiness indicators/tracer item at each facility level(number of facilities that have the tracer item available *100/ the total number of facilities); 2. Calculating the readiness index (RI) of facilities according to all 4 domains (the mean of all tracer item availability score in each domain);3. Calculating the facility level’s overall readiness score (the average of the readiness index of all 4 domains). Indices were stratified by facility level and compared to an RI cutoff score of 70%. This cutoff was based upon a study conducted by Wilbroad Mutale *et al* in Zambia which utilized the SARA tool, where scores above 70% were considered as ‘ready’ tomanage CRDs at that level (5). All analyses were performed using SPSS Vr 21.0.”

We have also deleted the paragraph describing MCWC,FWC in discussion.

Q3 addressed

Q4 There are still issues with syntax and grammar -see errors identified below

**Response:** Thank you for your feedback. We have addressed these issues mentioned below.

Line 68 health sector (singular)- **corrected in line 67 in the revised manuscript without track change mode**

Line 96 add ‘the’ WHO SARA standard tool- **corrected in line 96 in the revised manuscript without track change mode**
Line 98 – reduce ‘by’ one third- **corrected in line 98 in the revised manuscript without track change mode**
Line 110 – add ‘The’ WHO SARA standard tool—**corrected in line 110 in the revised manuscript without track change mode**
Line 118 – add hospitals ‘and health centres’—**corrected in line 125 in the revised manuscript without track change mode**
Line 124 and following – there is a reference only to hospitals eg ‘the head of each hospital’ – but the survey includes non hospital facilities – suggest replace the word hospital here with facility -**corrected in line 142 in the revised manuscript without track change mode**
Line 134 – ‘and enter data’ – add ‘to’ – and to enter data.—**corrected in line 146 in the revised manuscript without track change mode**
Line 173 – add ‘facility’ – only facility to cross---- **corrected in line 192 in the revised manuscript without track change mode**
Line 197 – replace ‘pick’ with ‘peak’ --- **corrected all over the manuscript, Table 1, Table 2 in the revised manuscript without track change mode**
Line 206 – replace ‘true’ with ‘found’—**corrected in line 222 in the revised manuscript without track change mode**
Line 221 - replace ‘few’ with ‘some’ and add ‘to this study’ – there are some limitations to this study---- **corrected in line 231 in the revised manuscript without track change mode**
Line 226 – separate Bangladeshis – to ‘Bangladesh is’--- **corrected in line 238 in the revised manuscript without track change mode**

Responses to comments
Comments on methods
(1) Additional information on facilities sampled:
The response does not address the issue of additional information on facilities except very briefly. It does not indicate the population served by each facility. Given the very large populations in Bangladesh, it is important that readers understand that a UHC serves a population greater than many districts in other countries. I would recommend a table as initially suggested with average populations per level of health facility.

**Response:** Thank you for your feedback. We have inserted the following paragraph in methods **line (114-120) in the revised manuscript without track change mode**

“In Bangladesh, public facilities are established according to its administrative units (division, district, upazila, union) where tertiary, secondary, and primary facilities are situated in the divisions (medical college hospitals), districts (DH, MCWC), upazila (UHC), union (FWC, CC) respectively [17]. As the number of population living in administrative units is different, so the population served by each facility is not defined. It may be mentioned that UHCs provide 31% of government health service and each of it serves 100,000 to 400,000 people depending on its size [18].”

Methods Table 2
This indicates the % of facilities in the sample, but does not indicate the total number of facilities in the country, so that the sample as a percentage of total facilities can be calculated. This data should be available from the main study. If so a reference to a published paper of the main study would be sufficient to cover this, although inclusion of the data would be preferable.
Line 119-123 states that a selection of facilities was made for the SARA parent study. Please state briefly how facilities were selected.

**Response:** Thank you for your feedback. We have included the required changes in methods like below **(line 121-137) in the revised manuscript without track change mode**

“To identify the total number of facilities at all three tiers in Bangladesh, the Bangladesh Health Facility Survey (BHFS) 2014, National Institute of Population Research and Training (NIPORT), Management Information System(MIS) Report of Directorate General of Health Services (DGHS), and records from director hospital administration DGHS were used (16). From this, we identified 19,184 total hospitals and health centers in the country [19] .

From this total, the parent study, Service Availability and Readiness Assessment (SARA)

Survey for NCDs and Disability Service Delivery System in Bangladesh, selected 590 facilities using a stratified random sampling procedure according to administrative units and level of facilities **(Fig1).** CRD services are provided from tertiary level of health facilities upto upazila level health facilities in Bangladesh. [8] This includes UHCs, district hospitals, and medical college hospitals. Conversely, MCWCs and FWCs were excluded from this sub study since these facilities only offer family planning services, and not CRD services. Therefore, this sub study found a total of 273 facilities that provided CRD services. However, 11 more facilities were excluded from this sub study during the analysis of data. These facilities were excluded either because the questionnaires were incomplete or the hospitals were specialized in diseases other than CRD and were therefore irrelevant for this current study. The resulting sample size of 262 facilities is depicted in **Fig1.**.

**Fig 1: Sampling and study inclusion flow chart**

Total no. of health facilities in Bangladesh:19,184

Total no. of health facilities in sample during survey: 590

- Tertiary hospital: 10
- District hospital (secondary): 60 (all)*
- MCWC (secondary): 61(all)
- UHC: 128 (30% of total UCHs in the country)
- FWC & CC:256 (2 facilities in correspondence to each UHC)
- NGO/Private: 75

No. of facilities excluded: 11

- Tertiary hospital: 2 (specialized in certain other disease like cancer and cardiovascular)
- District hospital:2 (incomplete questionnaire)
- UHC:4 (incomplete questionnaire)
- NGO/Private: 3 (incomplete questionnaire)

No. of facilities offered CRD services: 273

- Tertiary hospital: 10
- District hospital: 60
- UHC: 128
- NGO/Private: 75

No. of health facilities analyzed for this study: 262

- Tertiary hospital: 8
- District hospital: 58
- UHC: 124
- NGO/Private: 72

*N>B- UHC: Upazila Health Complex, MCWC: Maternal and Child Welfare Center, FWC: Family Welfare Center, CC: Community Clinic, NGO: Non Governmaent Organization.*

**rest of the district hospitals had been converted to medical college hospital (tertiary facilities)”*

**As the parent study is still not published, so we put the reference of the Bangladesh Health Facility Survey (BHFS) 2014 from where we collected the facility list.**

(2) Comments on results
This point has been adequately addressed
(3) Comments on discussion
See comments above on calculations of service readiness above. This comment has not been adequately addressed.

**Response:** Thank you so much for your feedback. We have changed the paragraph in discussion (excluded the portion of MCWC,FWC,CC). Also followed SARA tool calculating mean availability (table 2) and readiness (Fig 2,3)

Comment 2 has been adequately addressed
(4) Comments on conclusions
This comment was addressed in the limitations. However I note that although these changes are noted in the version of the report labelled ‘track changes’ – these changes are not included in the version of the report that appears to be the final revised version.
Table 1, although referred to in the revised version, is not included, although included in the track changes version.

**Response:** Thanks for addressing this issue. We have included these both in track change and clean version of the manuscript.

Corrections to syntax

**Response:**
Line 68 health sector (singular)- **corrected in line 67 in the revised manuscript without track change mode**

Line 96 add ‘the’ WHO SARA standard tool- **corrected in line 96 in the revised manuscript without track change mode**
Line 98 – reduce ‘by’ one third- **corrected in line 98 in the revised manuscript without track change mode**
Line 110 – add ‘The’ WHO SARA standard tool—**corrected in line 110 in the revised manuscript without track change mode**
Line 118 – add hospitals ‘and health centres’—**corrected in line 125 in the revised manuscript without track change mode**
Line 124 and following – there is a reference only to hospitals eg ‘the head of each hospital’ – but the survey includes non hospital facilities – suggest replace the word hospital here with facility -**corrected in line 142 in the revised manuscript without track change mode**
Line 134 – ‘and enter data’ – add ‘to’ – and to enter data.—**corrected in line 146 in the revised manuscript without track change mode**
Line 173 – add ‘facility’ – only facility to cross---- **corrected in line 192 in the revised manuscript without track change mode**
Line 197 – replace ‘pick’ with ‘peak’ --- **corrected all over the manuscript, Table 1, Table 2 in the revised manuscript without track change mode**
Line 206 – replace ‘true’ with ‘found’—**corrected in line 222 in the revised manuscript without track change mode**
Line 221 - replace ‘few’ with ‘some’ and add ‘to this study’ – there are some limitations to this study---- **corrected in line 231 in the revised manuscript without track change mode**
Line 226 – separate Bangladeshis – to ‘Bangladesh is’--- **corrected in line 238 in the revised manuscript without track change mode.**
